# Supplementary figures and images for: Ligand-specific regulation of transforming growth factor beta superfamily factors by leucine-rich repeats and immunoglobulin-like domains proteins
Source: PLoS One. 2023 Aug 21;18(8):e0289726. doi: 10.1371/journal.pone.0289726 (PMC10441800; doi:10.1371/journal.pone.0289726)

S1 Fig.

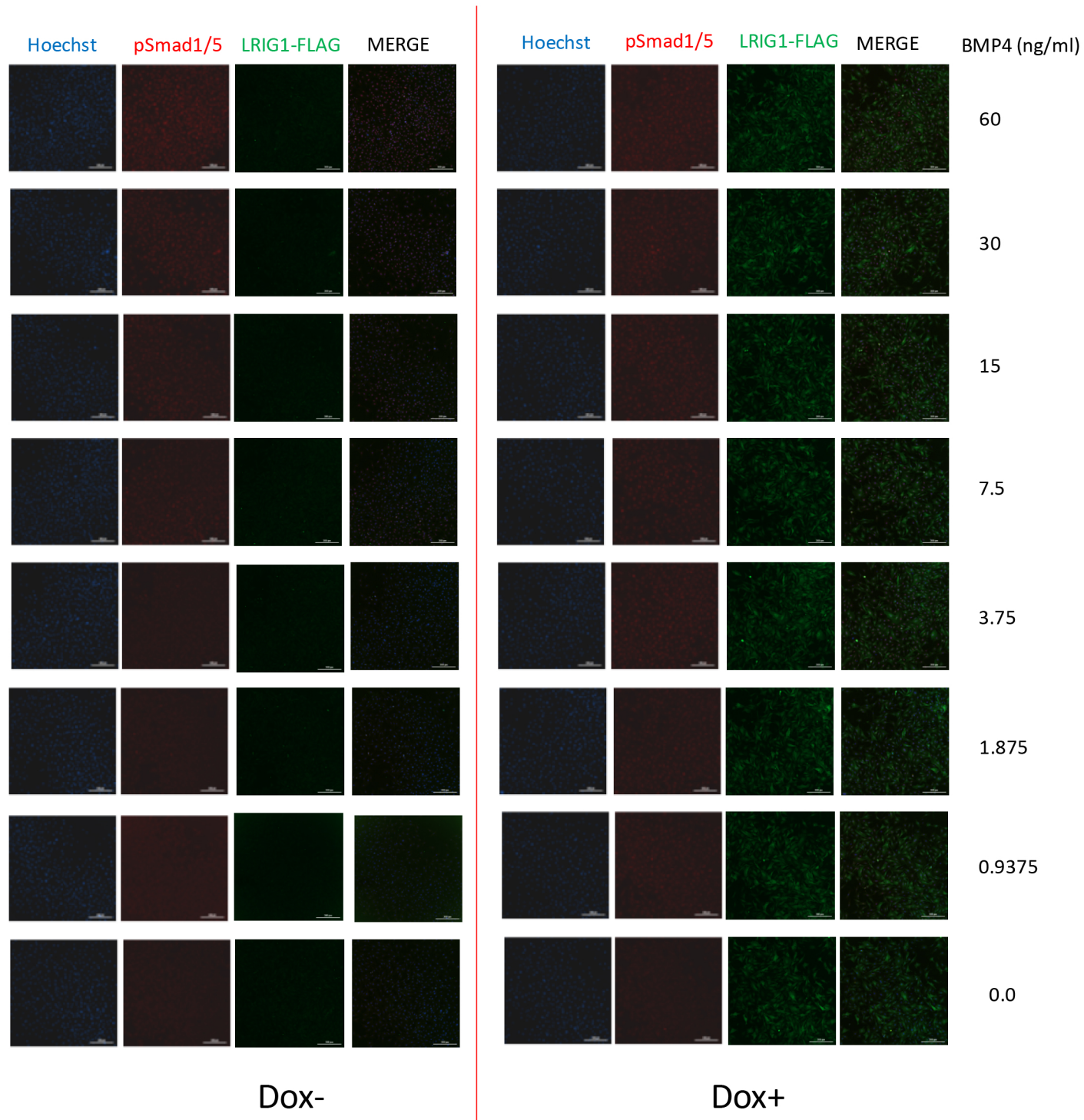

Dox-

Dox+

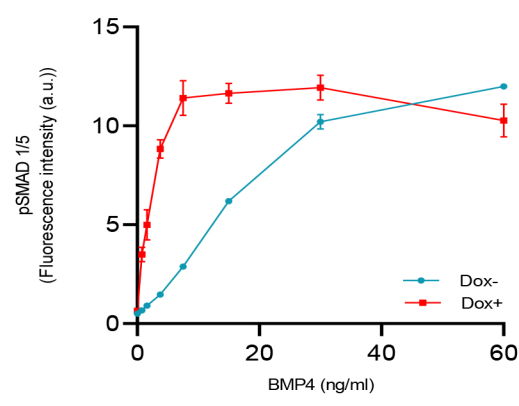

Supplement: S1 Fig — Alexa Fluor 647-labeled, nuclear localized pSmad1/5 was quantified and used as the readout for the activity of the BMP/GDF signaling branch. (PDF) [file pone.0289726.s001.pdf]

**S3 Fig.**

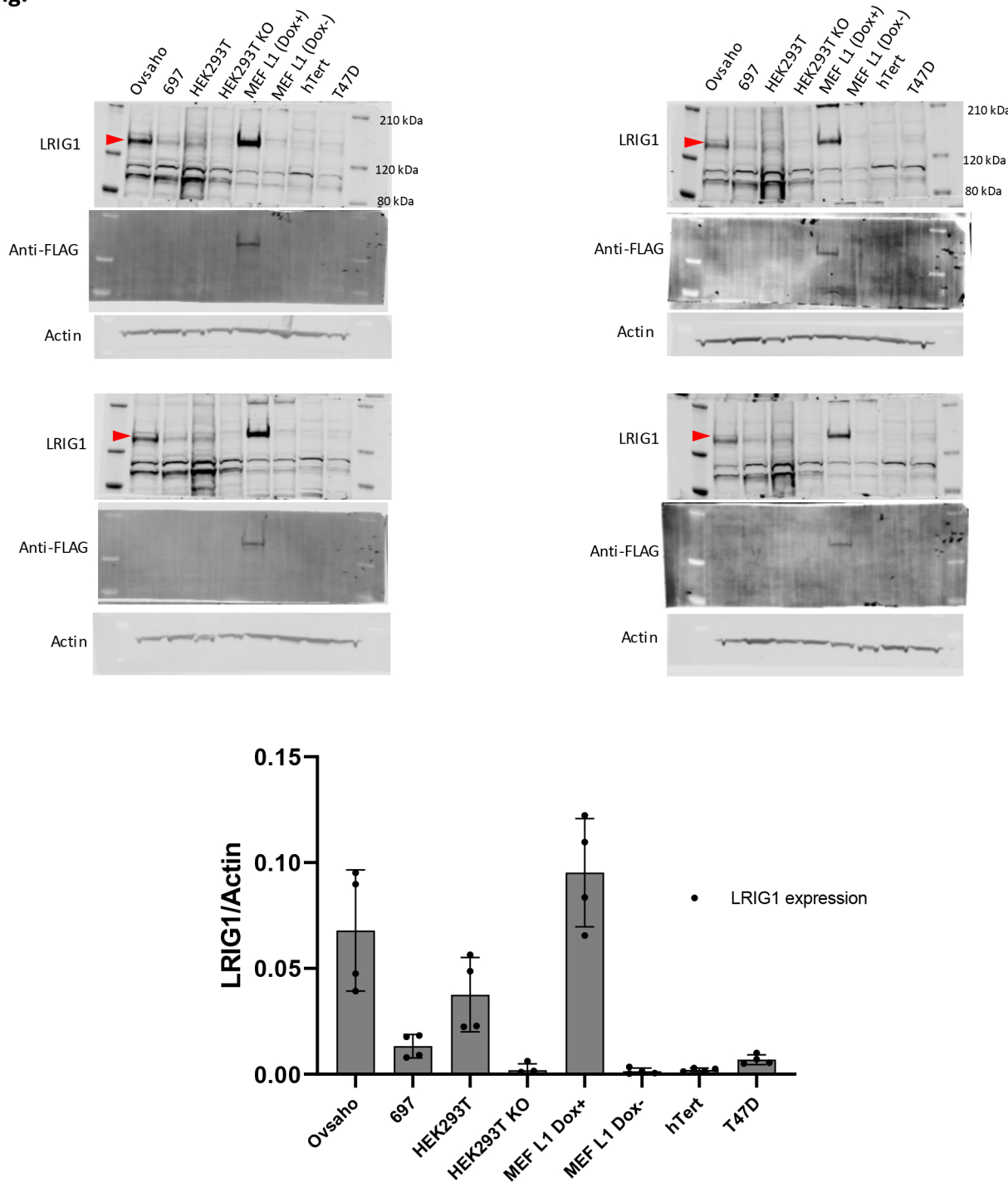

Supplement: S3 Fig — LRIG1 expression was induced by treating LRIG1-inducible MEFs with 100 ng/ml doxycycline for 24 hours (Dox+) or LRIG1 was not induced (Dox-). Respective cell cultures were lysed, and 5 μg of protein was analyzed for LRIG1 and actin by immunoblotting. The bar graphs show individual values, means, and standard deviations for LRIG1/actin ratios on an arbitrary scale from four independent experiments. There was no significant difference in the LRIG1/actin ratio between the LRIG1-induced MEFs and the human ovarian carcinoma cell line OVSAHO (p = 0.204, Student’s t test). (PDF) [file pone.0289726.s003.pdf]

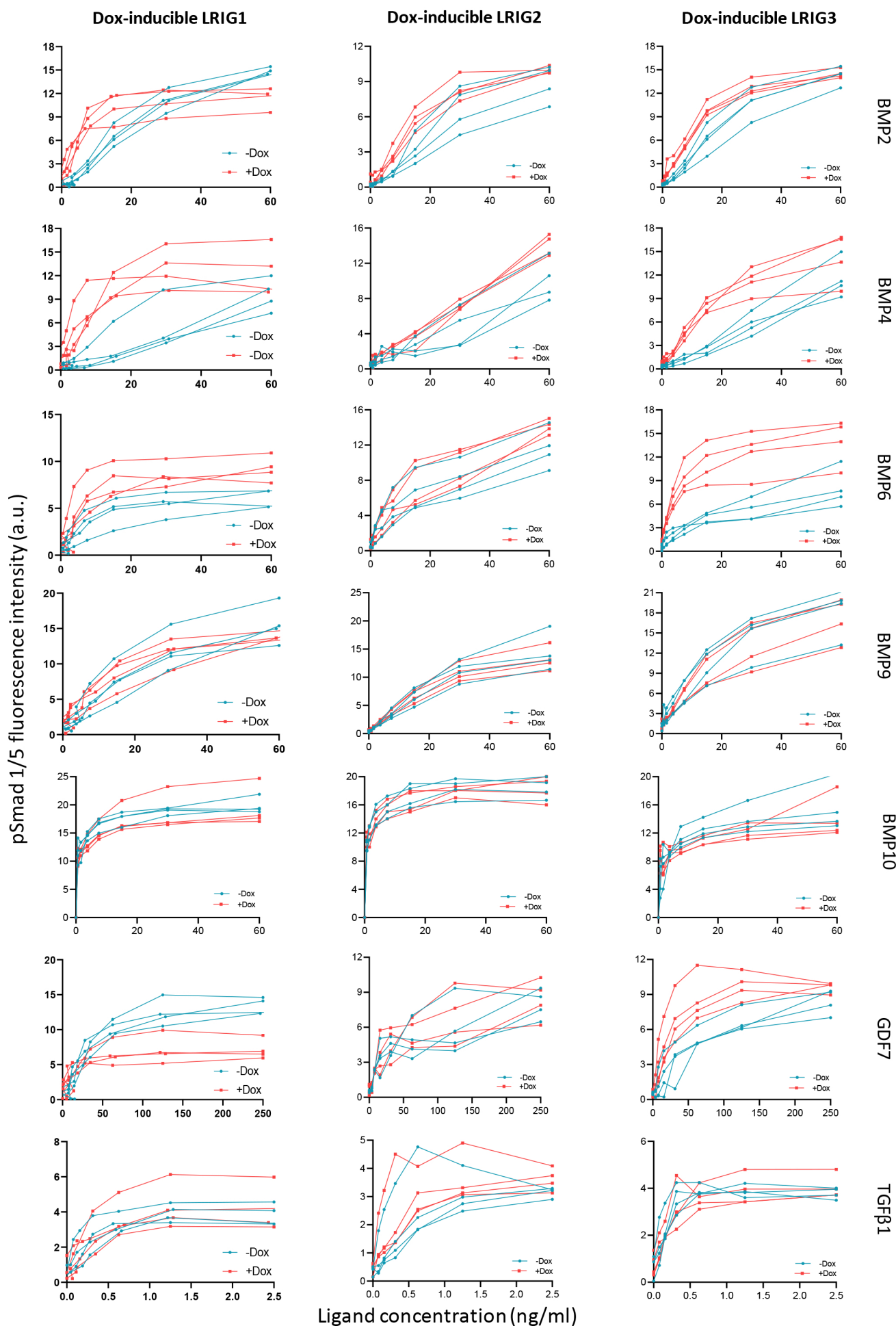

Supplement: S4 Fig — (PDF) [file pone.0289726.s004.pdf]

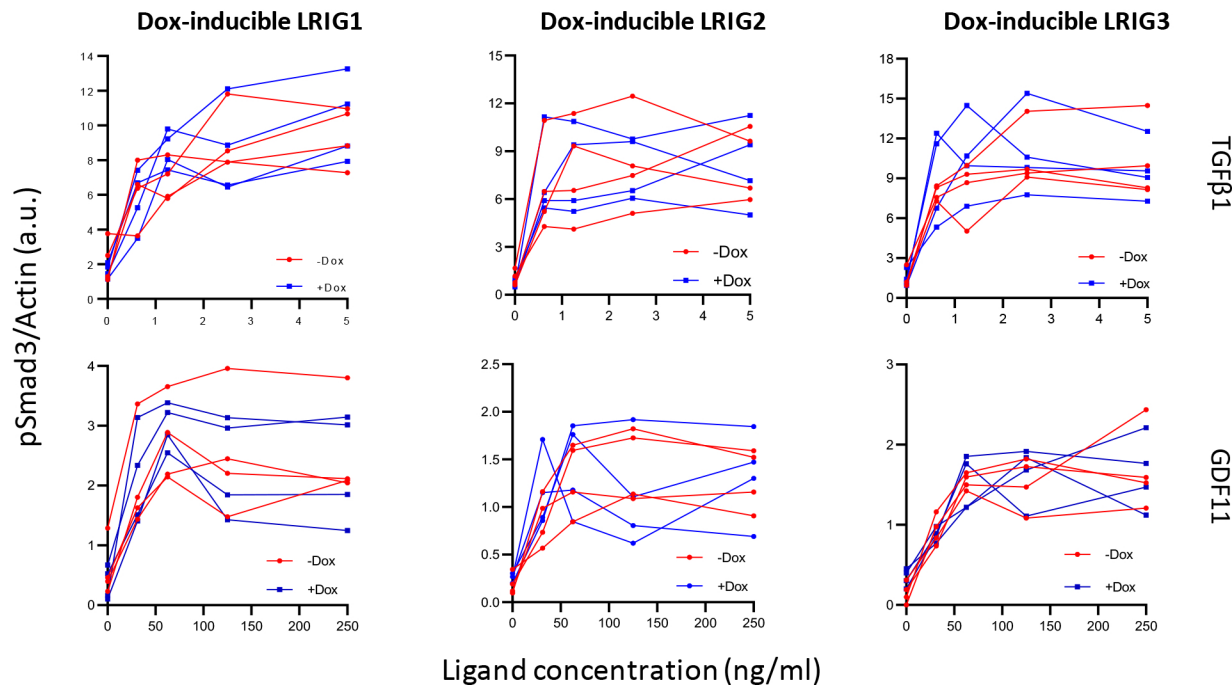

Supplement: S5 Fig — (PDF) [file pone.0289726.s005.pdf]

6 Fig.

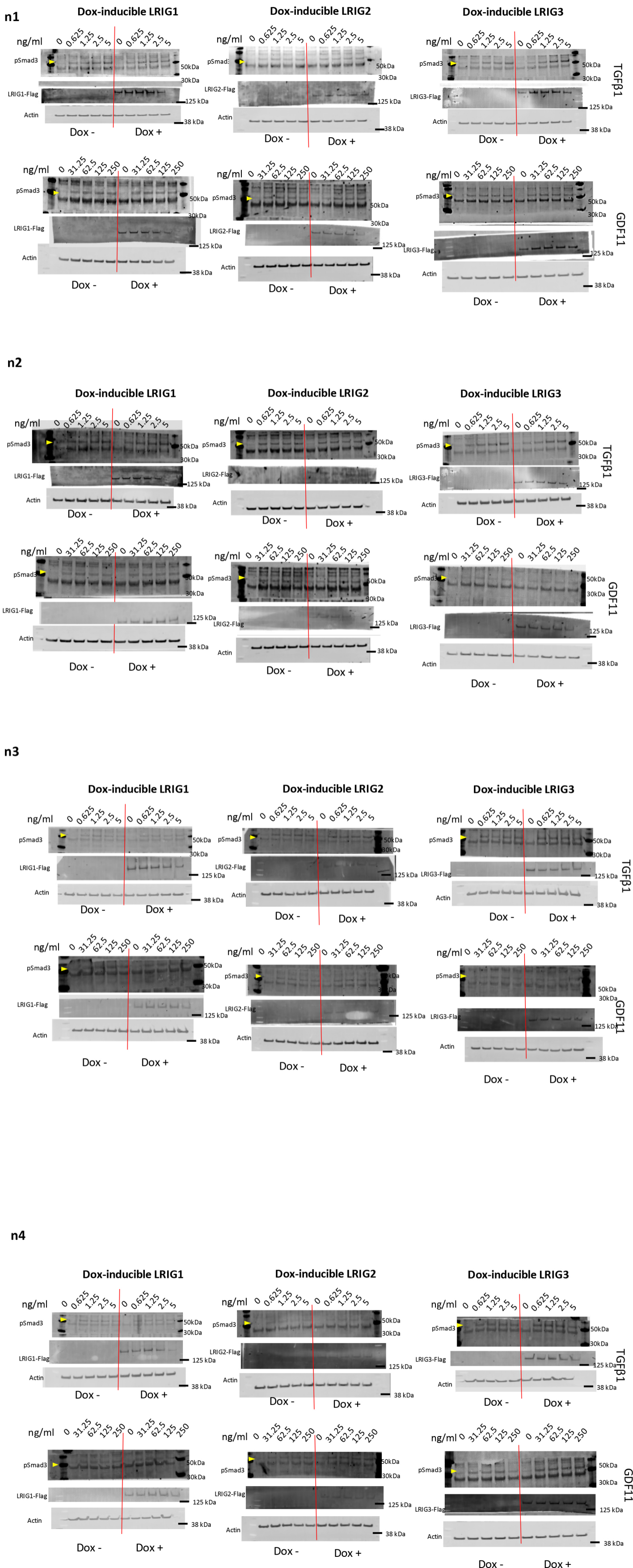

Supplement: S6 Fig — Phospho-Smad3 responses of Lrig-null MEFs with doxycycline-inducible LRIG1, LRIG2, or LRIG3 alleles to TGFβ1 and GDF11. LRIG1, LRIG2, or LRIG3 was induced by treating the respective cell line with 100 ng/ml doxycycline for 24 hours, followed by serum starvation and treatment with various concentrations of TGFβ1 or GDF11 for 1 hour. (PDF) [file pone.0289726.s006.pdf]

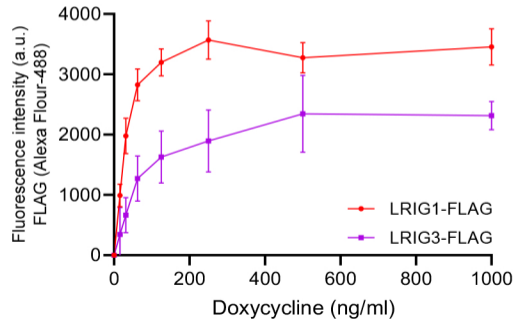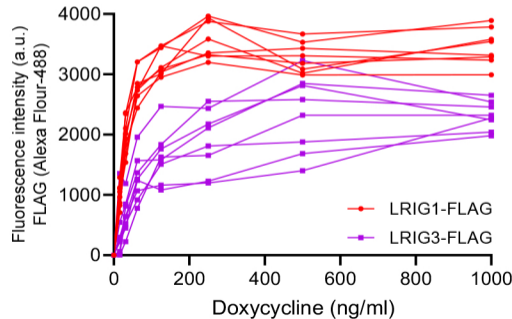

Supplement: S7 Fig — (PDF) [file pone.0289726.s007.pdf]

pSmad 1/5 fluorescence intensity (a.u.)

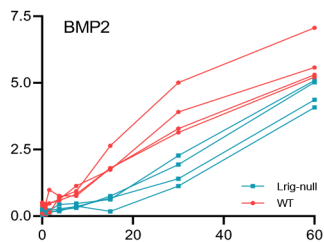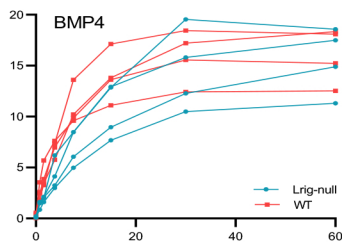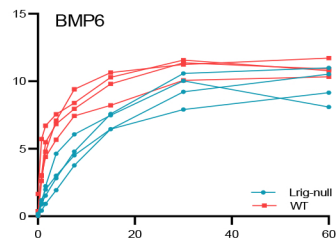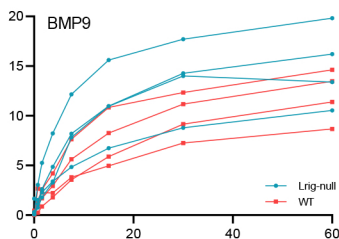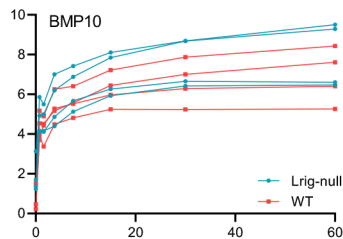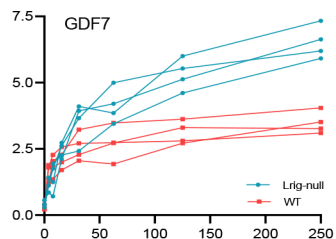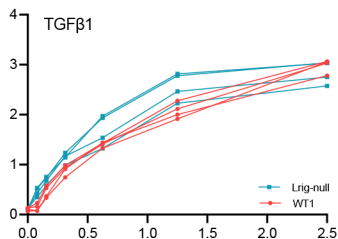

Ligand concentration (ng/ml)

Supplement: S8 Fig — (PDF) [file pone.0289726.s008.pdf]

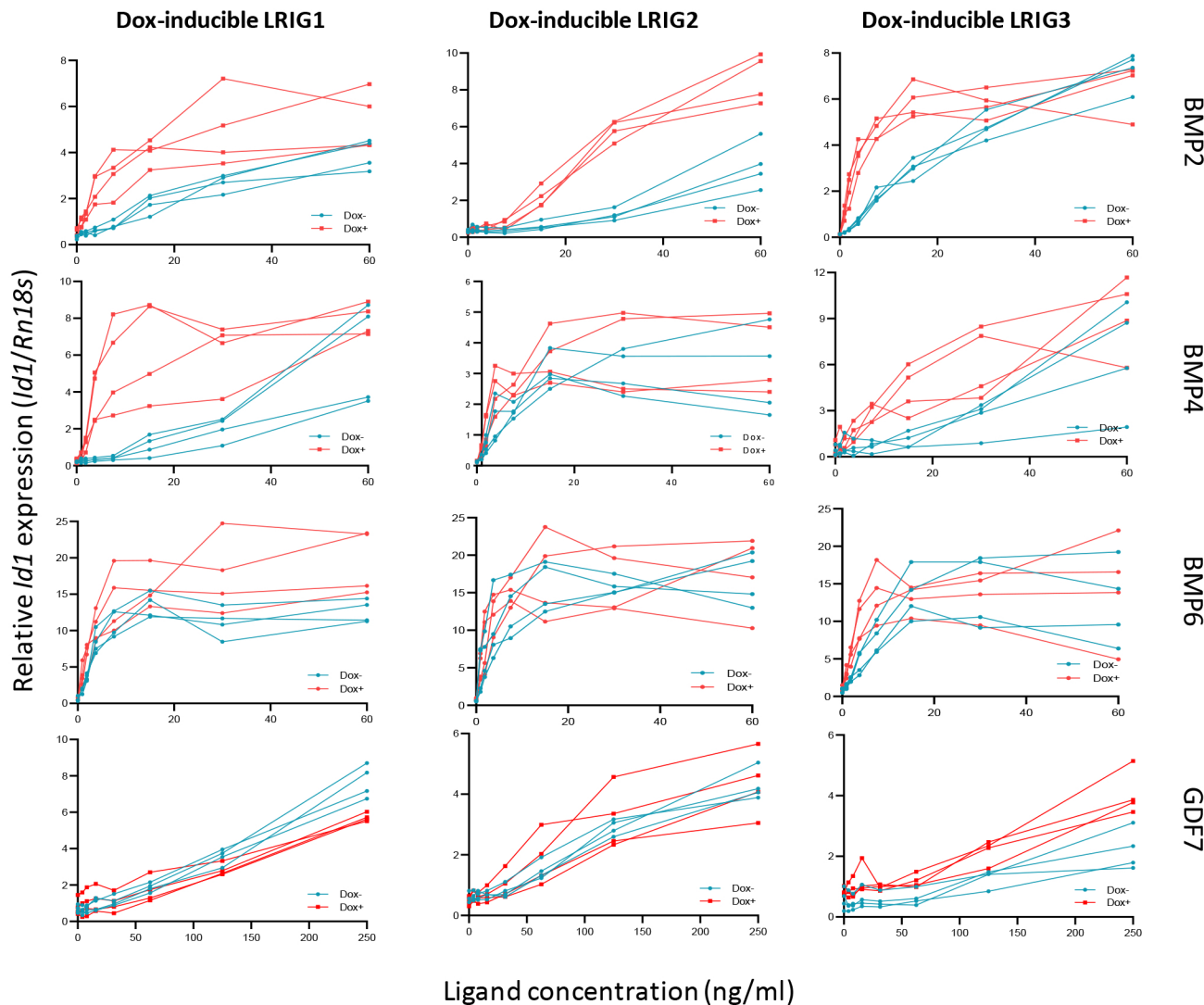

Supplement: S9 Fig — (PDF) [file pone.0289726.s009.pdf]
