# Supplementary material for: Ligand-specific regulation of transforming growth factor beta superfamily factors by leucine-rich repeats and immunoglobulin-like domains proteins
Source: PLoS One. 2023 Aug 21;18(8):e0289726. doi: 10.1371/journal.pone.0289726 (PMC10441800; doi:10.1371/journal.pone.0289726)
Supplement: S1 Table — (PDF) [file pone.0289726.s010.pdf]

S1 Table. TGFβ family ligands used in this study.

Ligand	Vendor	Cat.no.	Lot no.	ED_{50}[a] (ng/ml)	Assay[b]
BMP2	Peprotech[c]	120-02C	1015595	40-100	ALP[d] production by ATDC-5 cells.
BMP3	Peprotech	120-24B	0710523	Not indicated	
BMP4	Peprotech	120-05	0421526	2-8	ALP production by ATDC-5 cells.
BMP6	Peprotech	120-06	unknown	2-8	pSmad1/5 response in MEFs [15].
BMP9/GDF2	Peprotech	120-07	0909495	0.5–1.9	ALP production by ATDC-5 cells.
BMP10	Peprotech	120-40	0912550	4.0-6.0	ALP production by ATDC-5 cells.
BMP15	R&D Systems[e]	5096-BM	RDE2222061	10	Smad3 phosphorylation in P19 mouse embryonal carcinoma cells.
Activin A	Peprotech	120-14E	1115478-1	≤ 2.0	Inhibition of the proliferation of MPC-11 cells.
GDF3	Peprotech	120-22	0907326	100-150	Inhibition of induced ALP production by ATDC-5 chondrogenic cells.
GDF7	Peprotech	120-37	1210528	Not indicated	
GDF11	Peprotech	120-11	0917295	1. 80-100<br>2. 8-10	1. Inhibition of induced ALP production by ATDC-5 cells.<br>2. Inhibition of ALP activity in differentiating MC3T3/E1 cells
GDF15	Peprotech	120-28C	0316552	75-200	Inhibition of ALP activity in differentiating MC3T3/E1 osteoblast cells.
TGFβ1	Peprotech	100-21	05212209	0.05	Inhibition of IL-4-dependent proliferation of HT-2 cells.

[a]Median effective dose (ED_{50}) indicated in the vendor's data sheet or in publication.
[b]Assay used by vendor or investigator to determine the ED_{50}.
[c]PeproTech Nordic, Stockholm, Sweden
[d]ALP, alkaline phosphatase
[e]R&D Systems, Minneapolis, MN, USA
