# Supplementary material for: Ligand-specific regulation of transforming growth factor beta superfamily factors by leucine-rich repeats and immunoglobulin-like domains proteins
Source: PLoS One. 2023 Aug 21;18(8):e0289726. doi: 10.1371/journal.pone.0289726 (PMC10441800; doi:10.1371/journal.pone.0289726)
Supplement: S2 Table — (PDF) [file pone.0289726.s011.pdf]

S2 Table. Antibodies used in this study.

Antigen and antibody conjugate	Host species and antibody clonality[a]	Vendor	Cat. no.	Lot no.	Application[b]	Dilution
pSmad1/5	Rabbit mAb	Cell Signaling Technology	9516	10	IB, IF	1:1,000 (WB) 1:800 (IF)
pSmad3	Rabbit mAb	Cell Signaling Technology	9520	16	IB	1:1,000
LRIG1	Rabbit pAb	In-house	n/a	Carmener 3448U	IB	1 µg/ml
FLAG M2	Mouse mAb	Sigma–Aldrich	F3165	SLCC4005	IB, IF	1:20,000 (WB) 1:2,000 (IF)
Actin	Mouse mAb	Cell Signaling Technology	3700	18	IB	1:5,000
Mouse IgG IRDye 800CW	Goat pAb	LI-COR Biosciences	926-32210	C81106-03	IB	1:15,000
Rabbit IgG IRDye 680RD	Goat pAb	LI-COR Biosciences	925-68071	D00115-06	IB	1:15,000
Rabbit IgG Alexa fluor 647	Goat pAb	Invitrogen	A21245	2232862	IF	1:1,000
Mouse IgG Alexa fluor 488	Donkey pAb	Invitrogen	A21202	2266877	IF	1:1,000

[a]mAb, monoclonal antibody; pAb, polyclonal antibody

[b]IB, immunoblotting; IF, immunofluorescence
