# Supplementary material for: Ligand-specific regulation of transforming growth factor beta superfamily factors by leucine-rich repeats and immunoglobulin-like domains proteins
Source: PLoS One. 2023 Aug 21;18(8):e0289726. doi: 10.1371/journal.pone.0289726 (PMC10441800; doi:10.1371/journal.pone.0289726)
Supplement: S3 Table — (PDF) [file pone.0289726.s012.pdf]

**S3 Table. Oligonucleotide primer and probe sequences for quantitative real-time RT–PCR.**

| Templates | Oligonucleotides | Sequences[a] (5'→3') | Vendor |
|---|---|---|---|
| *Lrig1* exon 1 | Forward primer | TTGCGCCTCGCCTTCTGCTC | MWG[b] |
| | Reverse primer | CCACTGCAGTCCAGCGAGTCC | MWG[b] |
| | Probe | fCTGCTACQGCAATGGCCGGAGTCAGCGG | SGS[c] |
| *Lrig2* exon 12-13 | Forward primer | TGGCTGGTGGATAACAATTTCCATCATTCT | SGS[c] |
| | Reverse primer | TCTGAGGCTTCAGAAAATCATCACAGAC | SGS[c] |
| | Probe | fCACATCCQGAATGGTTAGCGGGGCAAAGCATCTTGAATG | SGS[c] |
| *RN18S* | Forward primer | CGGCGACGACCCATTCGAAC | IDT[d] |
| | Reverse primer | GAATCGAACCCTGATTCCCCGTC | IDT[d] |
| | Probe | fCCTATCAACQTTCGATGGTAGTCGCCGTGCC | IDT[d] |

[a]f denotes a 5’-conjugated fluorescein, and Q denotes a T with a conjugated dark quencher.
[b]MWG Biotech AG, Ebersberg, Germany
[c]Scandinavian Gene Synthesis AB, Köping, Sweden
[d]Integrated DNA Technologies (IDT), Leuven, Belgium
