# Supplementary material for: Ligand-specific regulation of transforming growth factor beta superfamily factors by leucine-rich repeats and immunoglobulin-like domains proteins
Source: PLoS One. 2023 Aug 21;18(8):e0289726. doi: 10.1371/journal.pone.0289726 (PMC10441800; doi:10.1371/journal.pone.0289726)
Supplement: S4 Table — (PDF) [file pone.0289726.s013.pdf]

S4 Table. Gene expression data[a] for TGFβ family receptors and coreceptors and Lrig proteins in wild-type and *Lrig*-null MEFs.

		Wild-type MEFs		*Lrig*-null MEFs	
Receptor type	Gene	Reads[b]	S.D.[c]	Reads[b]	S.D.[c]
Type 1 receptors					
	Tgfbr1	674	129	686	163
	Acvr1	1,635	161	1,410	108
	Acvr1b	458	59	401	52
	Acvr1c	0.8	0.8	0.2	0.5
	Acvrl1	374	52	421	55
	Bmpr1a	3,512	375	3,730	500
	Bmpr1b	110	15	94	30
Type 2 receptors					
	Tgfbr2	2,672	115	3,076	591
	Acvr2a	304	46	319	64
	Acvr2b	68	25	66	14
	Bmpr2	3,748	1,094	3,676	1,143
	Amhr2	4	1	4	4
Coreceptors					
	Eng/Endoglin	86	8	74	11
	Tgfbr3/Betaglycan	516	87	627	195
	Rgma	538	67	622	137
	Rgmb	493	97	538	108
	Rgmc/*Hfe2*	0	0	0	0
	Ror1	16	4	18	5
	Ror2	246	56	152	52
	Neo1	7,814	1,406	7,108	1,147
	Ntn1	720	95	638	136
LRIGs					
	Lrig1	2,566	349	1,300	281
	Lrig2	665	72	151	17
	Lrig3	1,034	108	4	1

[a]Dataset was retrieved from NCBI BioProject repository, https://www.ncbi.nlm.nih.gov/bioproject/PRJNA684140 [15].

[b]Apparent number of RNAseq reads. The means of four different wild-type cell lines and four different *Lrig*-null cell lines are shown.

[c]Standard deviations (S.D.) of the four cell lines.
