# Supplementary material for: Ligand-specific regulation of transforming growth factor beta superfamily factors by leucine-rich repeats and immunoglobulin-like domains proteins
Source: PLoS One. 2023 Aug 21;18(8):e0289726. doi: 10.1371/journal.pone.0289726 (PMC10441800; doi:10.1371/journal.pone.0289726)
Supplement: S5 Table — (PDF) [file pone.0289726.s014.pdf]

S5 Table. Gene expression data (qRT–PCR) for Lrig genes in wild-type and *Lrig*-null MEFs.

	Wild-type MEFs		*Lrig*-null MEFs	
Gene	Mean level[a]	S.D[b]	Mean level[a]	S.D[b]
Lrig1	54.2	8.4	1.6	1.8
Lrig2	35.2	5.8	3.0	2.9
Lrig3	30.1	5.8	2.3	2.2

[a]Mean relative gene expression levels of the four wild-type and four *Lrig*-null MEF lines, determined through qRT–PCR using primers and probes that were specific for the ablated region of the respective gene. The numbers indicate the *Lrig*/*Rn18s* ratio in arbitrary units.[b]Standard deviations (S.D.) of the four cell lines.
