# Supplementary material for: Ligand-specific regulation of transforming growth factor beta superfamily factors by leucine-rich repeats and immunoglobulin-like domains proteins
Source: PLoS One. 2023 Aug 21;18(8):e0289726. doi: 10.1371/journal.pone.0289726 (PMC10441800; doi:10.1371/journal.pone.0289726)
Supplement: S1 Raw images — (PDF) [file pone.0289726.s016.pdf]

Uncropped blots of Supplementary Figure 2

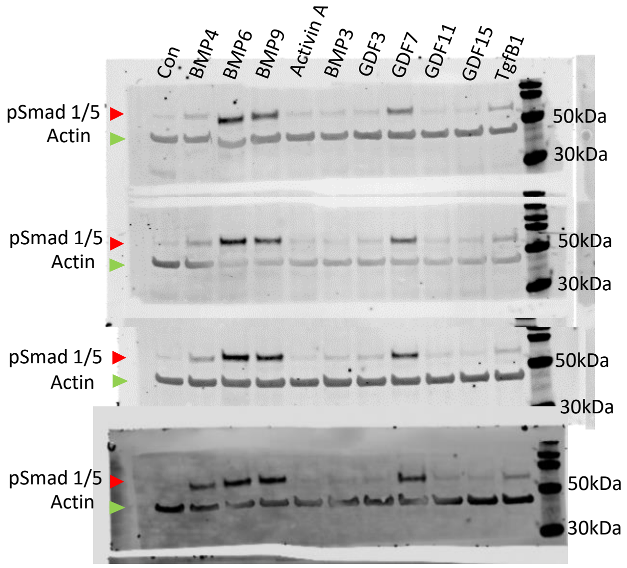

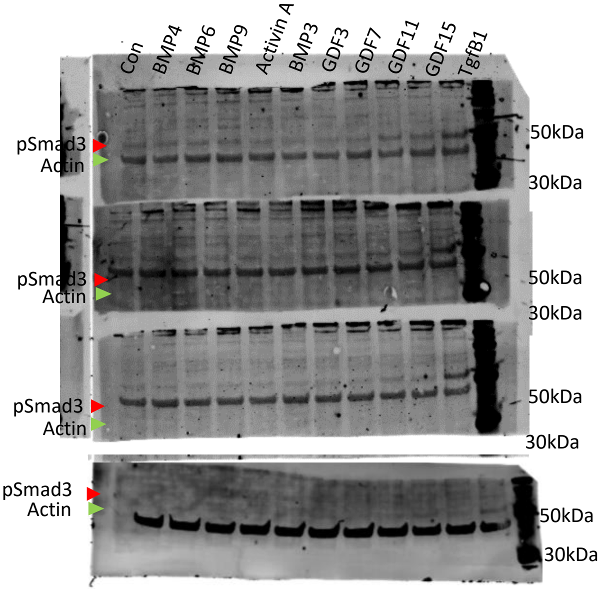

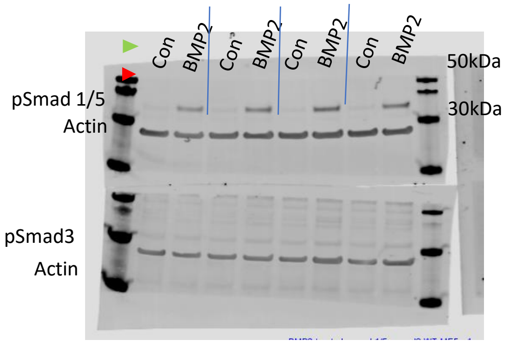

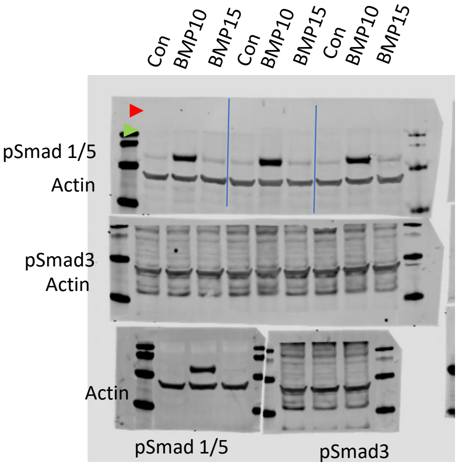

Uncropped blots of Supplementary Figure 3

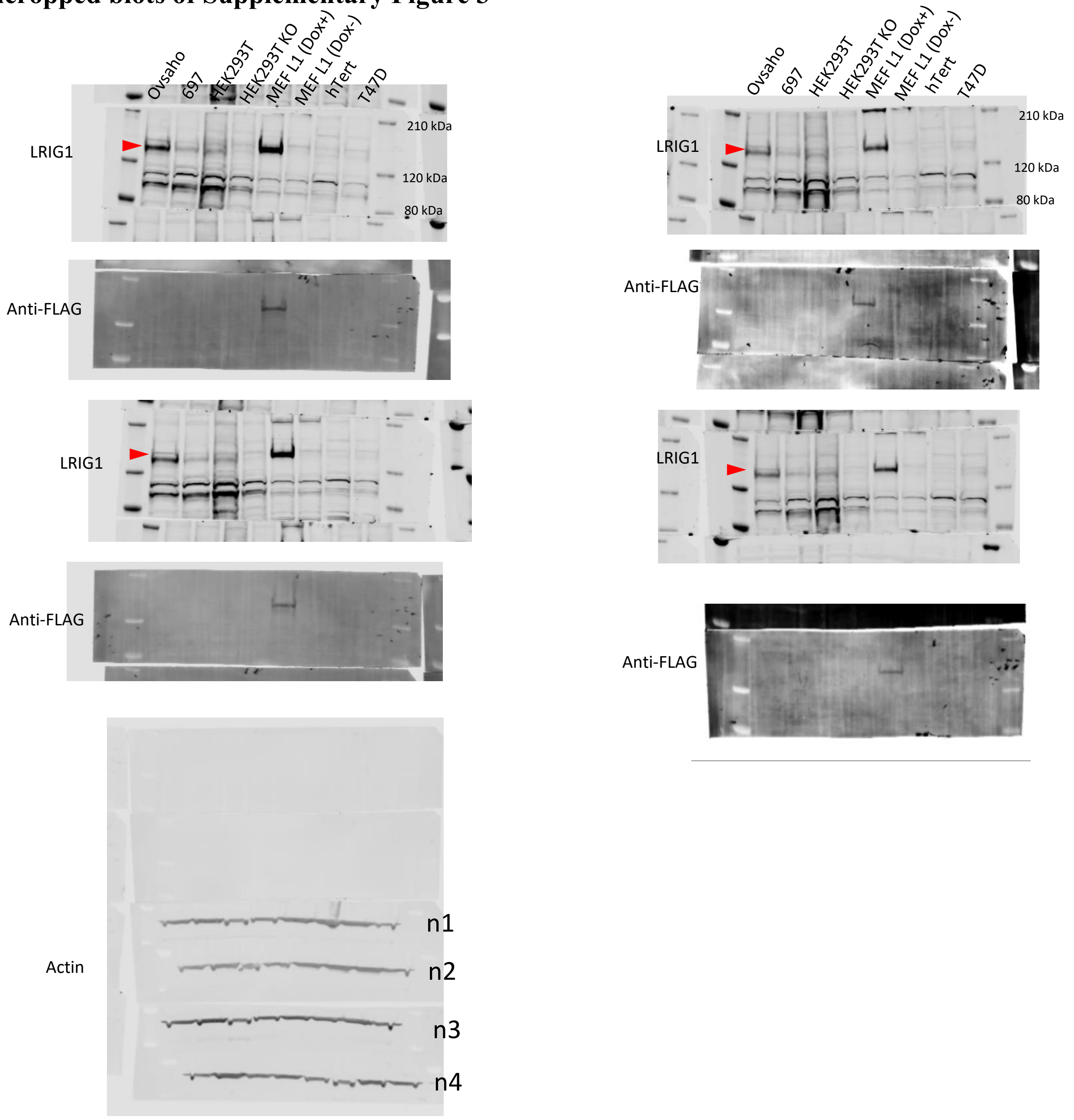

Uncropped blots of Supplementary Figure 6

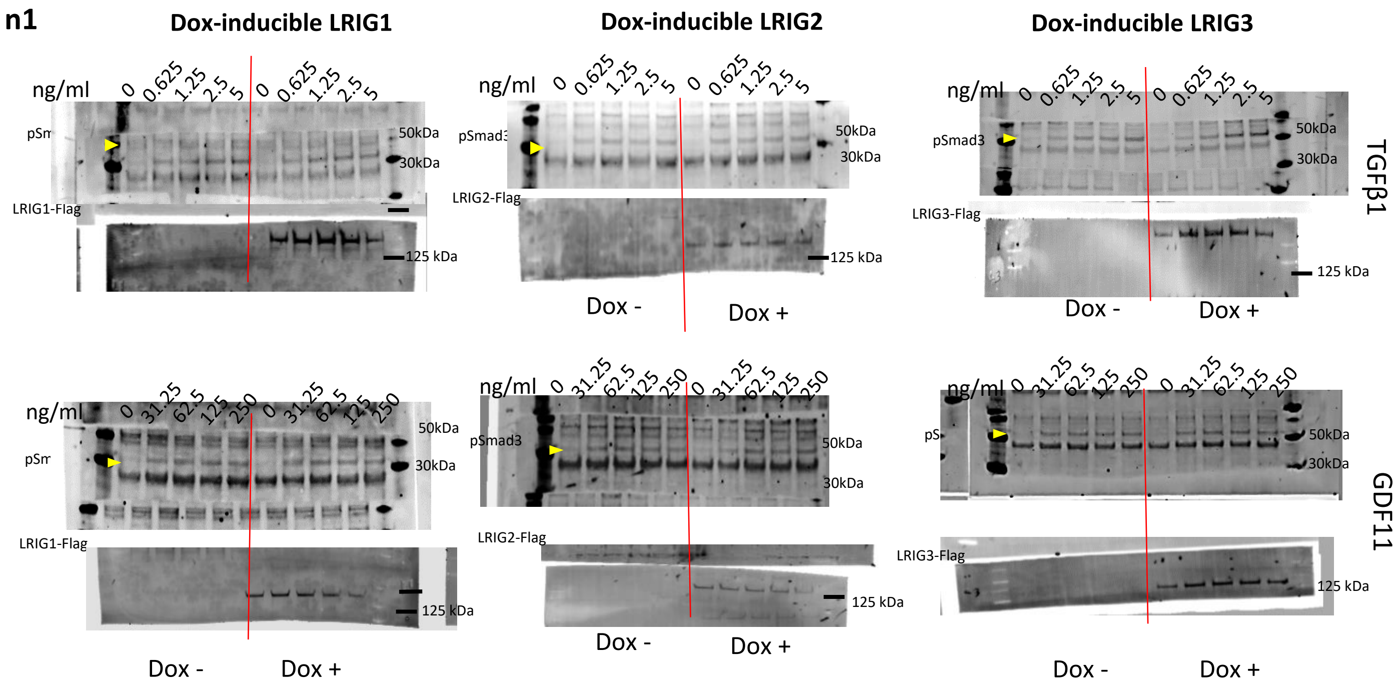

Actins

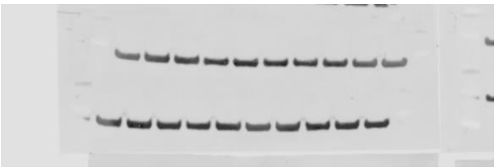

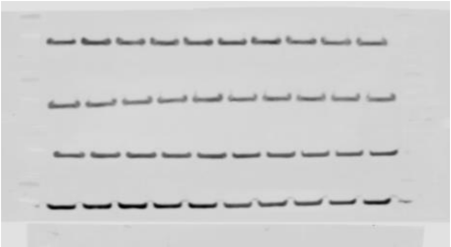

n2

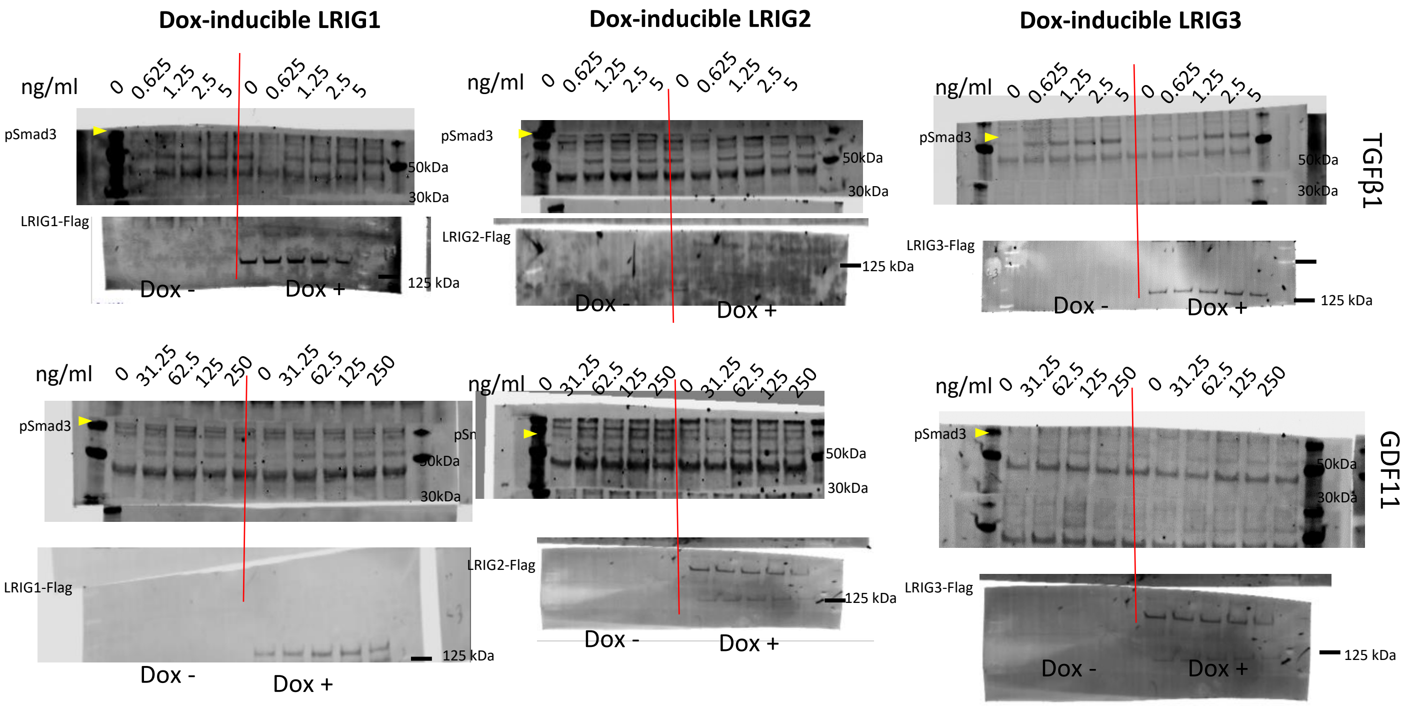
Dox-inducible LRIG1
Dox-inducible LRIG2
Dox-inducible LRIG3
ng/ml
0 0.625 1.25 2.5 5 0 0.625 1.25 2.5 5
0 31.25 62.5 125 250 0 31.25 62.5 125 250
pSmad3
50kDa
30kDa
LRIG1-Flag
LRIG2-Flag
LRIG3-Flag
125 kDa
Dox -
Dox +
TGFβ1
GDF11

Actins

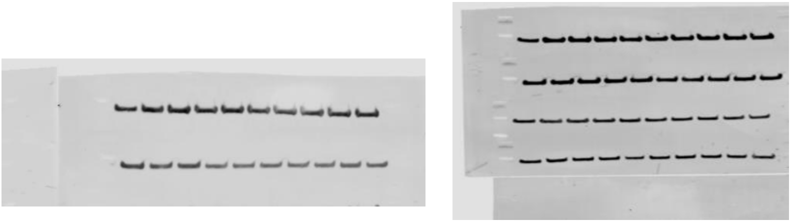

n3

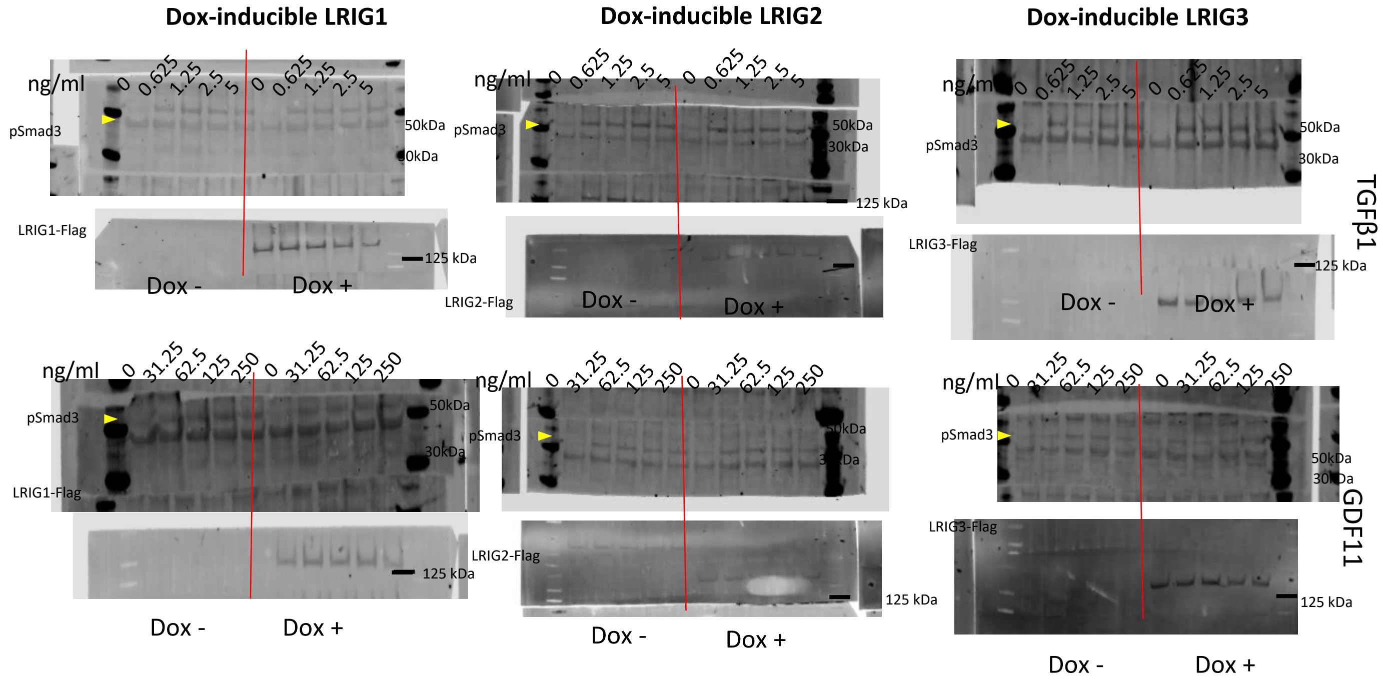
Dox-inducible LRIG1
Dox-inducible LRIG2
Dox-inducible LRIG3
ng/ml
0
0.625
1.25
2.5
5
31.25
62.5
125
250
pSmad3
LRIG1-Flag
LRIG2-Flag
LRIG3-Flag
50kDa
30kDa
125 kDa
Dox -
Dox +
TGFβ1
GDF11

Actins

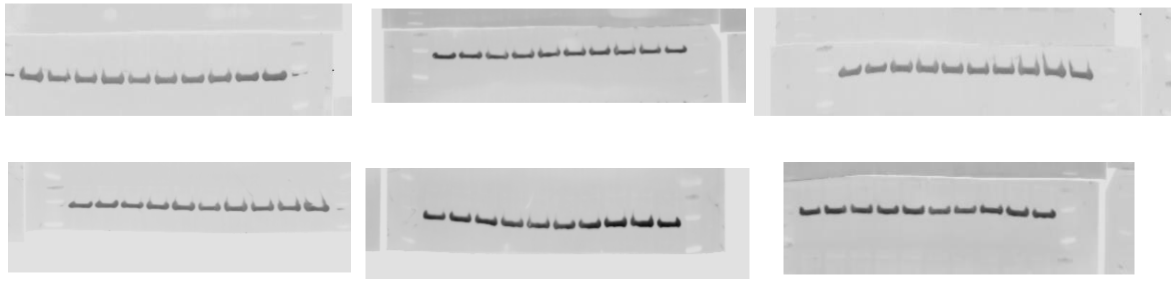

n4

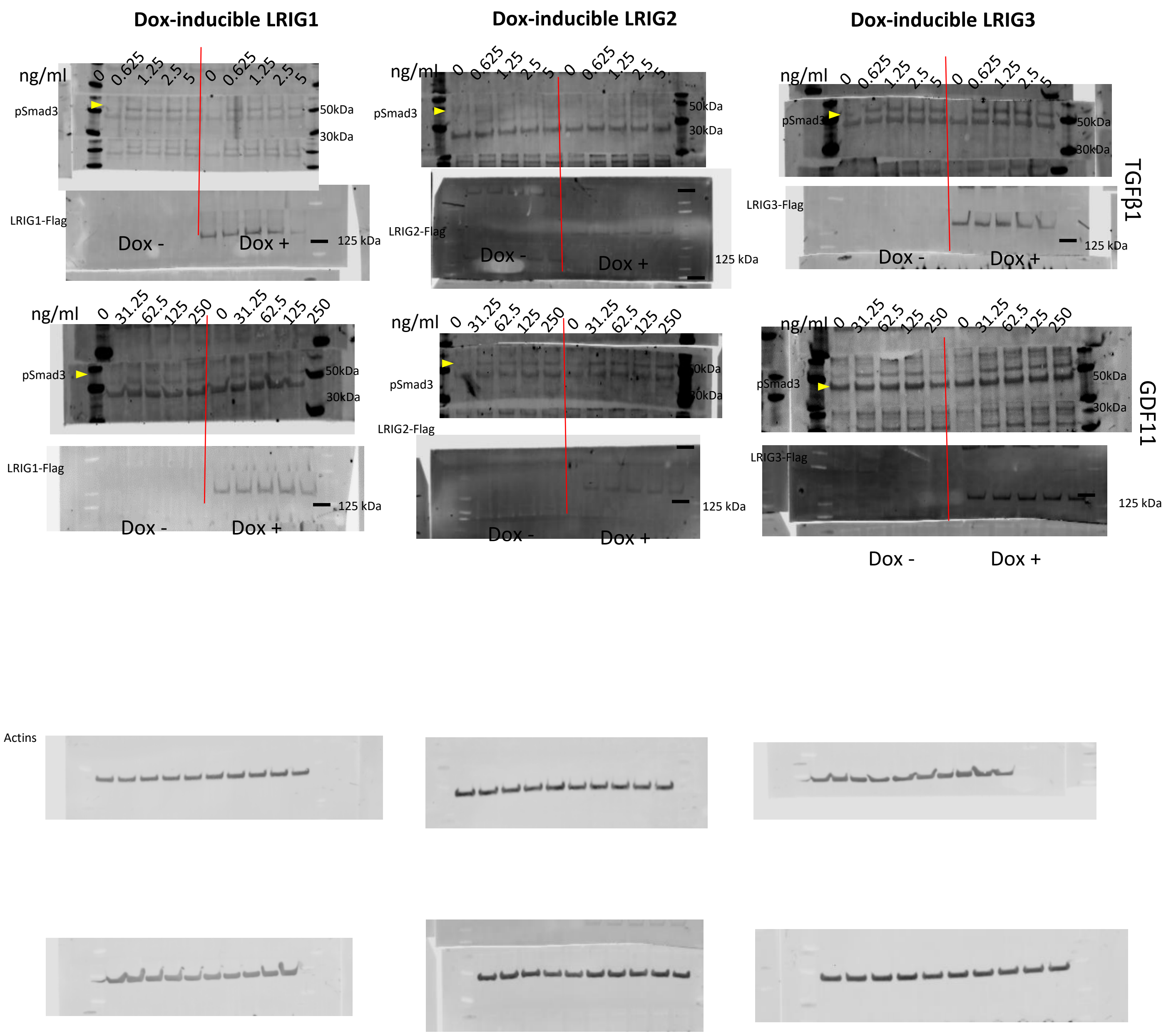

Dox-inducible LRIG1
Dox-inducible LRIG2
Dox-inducible LRIG3
ng/ml
0
0.625
1.25
2.5
5
pSmad3
50kDa
30kDa
LRIG1-Flag
LRIG2-Flag
LRIG3-Flag
Dox -
Dox +
125 kDa
TGFβ1
31.25
62.5
125
250
GDF11
Actins
