# Supplementary material for: Ligand-specific regulation of transforming growth factor beta superfamily factors by leucine-rich repeats and immunoglobulin-like domains proteins
Source: PLoS One. 2023 Aug 21;18(8):e0289726. doi: 10.1371/journal.pone.0289726 (PMC10441800; doi:10.1371/journal.pone.0289726)
Supplement: S2 Fig — Thirteen TGFβ family ligands, representing all the major TGFβ family subgroups, were evaluated for their ability to induce the phosphorylation of Smad1/5 (pSmad1/5) or Smad3 (pSmad3) in wild-type MEFs at their ED50. Ligands and the concentrations used were as follows: BMP2, 10 ng/ml; BMP3, 100 ng/ml; BMP4, 10 ng/ml; BMP6, 6 ng/ml; BMP9, 2 ng/ml; BMP10, 6 ng/ml; BMP15, 10 ng/ml, Activin A, 2 ng/ml; GDF3, 150 ng/ml; GDF7, 100 ng/ml; GDF11, 100 ng/ml; GDF15, 200 ng/ml; and TGFβ1, 0.5 ng/ml. Relative pSmad1/5, pSmad3, and actin levels were quantified through immunoblotting. The bar graphs show the mean pSmad/actin ratios of four independent experiments, with error bars indicating the standard deviations. (Significant differences compared with the untreated control cells are indicated with asterisks: *p < 0.05; **p < 0.01; ***p < 0.001; ****p < 0.0001, Student’s t test). (PDF) [file pone.0289726.s002.pdf]

S2 Fig.

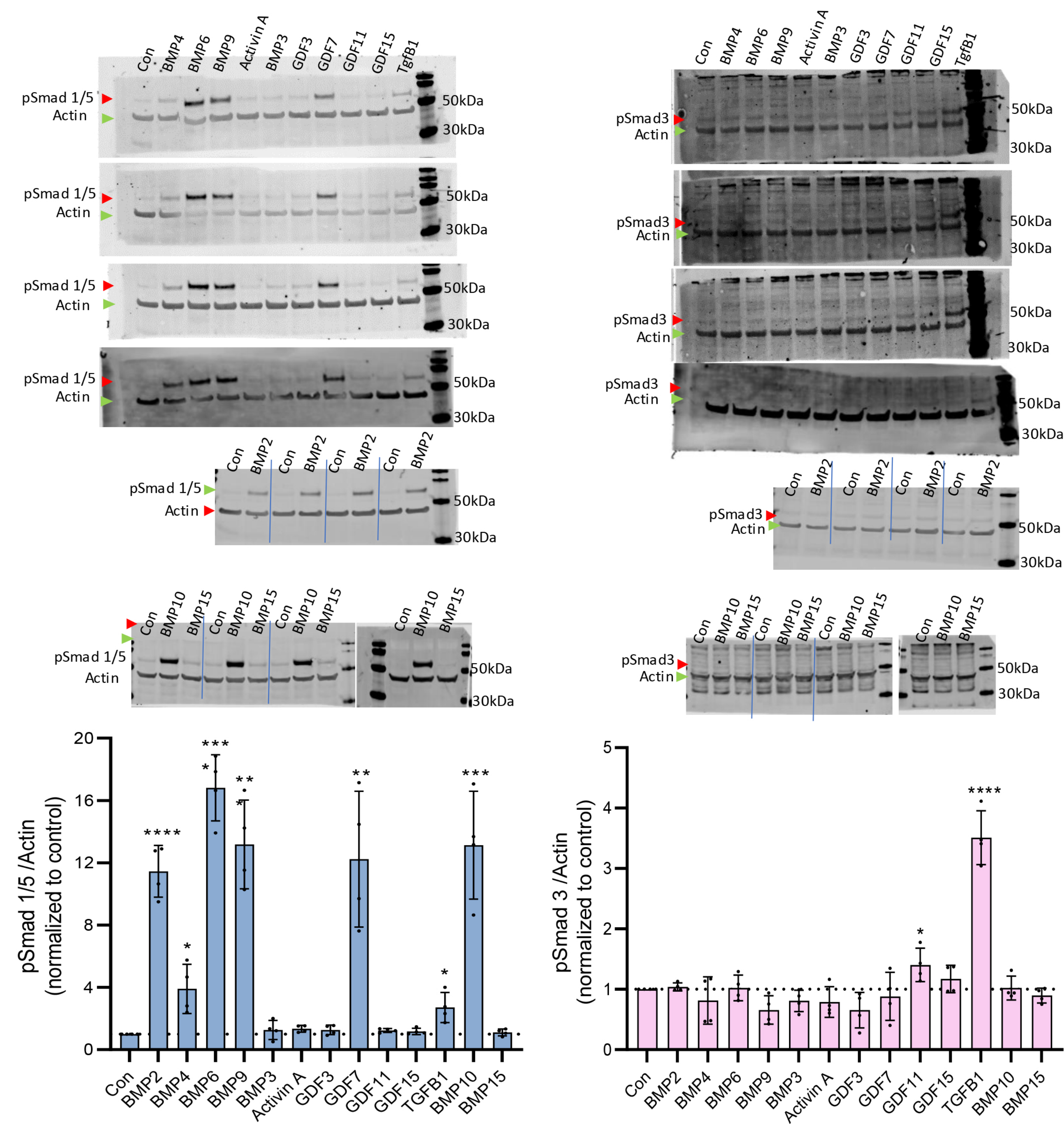
Con BMP4 BMP6 BMP9 Activin A BMP3 GDF3 GDF7 GDF11 GDF15 TgfB1
pSmad 1/5
Actin
50kDa
30kDa
pSmad3
Actin
Con BMP2 Con BMP2 Con BMP2 Con BMP2
Con BMP10 BMP15 Con BMP10 BMP15 Con BMP10 BMP15 Con BMP10 BMP15
pSmad 1/5 /Actin (normalized to control)
pSmad 3 /Actin (normalized to control)
Con BMP2 BMP4 BMP6 BMP9 BMP3 Activin A GDF3 GDF7 GDF11 GDF15 TGFB1 BMP10 BMP15
