# Supplementary material for: Ligand-specific regulation of transforming growth factor beta superfamily factors by leucine-rich repeats and immunoglobulin-like domains proteins
Source: PLoS One. 2023 Aug 21;18(8):e0289726. doi: 10.1371/journal.pone.0289726 (PMC10441800; doi:10.1371/journal.pone.0289726)
Supplement: Attachment — Submitted filename: Rebuttal letter 230602.docx [file pone.0289726.s017.docx]

Rebuttal letter

Dear Dr. Bernard and Referees,

Thank you for critically reviewing our manuscript PONE-D-23-05403, titled "Ligand-Specific Regulation of Transforming Growth Factor Beta Superfamily Factors by Leucine-rich Repeats and Immunoglobulin-like Domains Proteins”. We appreciate the reviewers’ comments, which were well taken and have improved the manuscript. Attached is the revised version of the manuscript. All changes made to the manuscript can be viewed in the tracked changes in the attached Word file. A point-by-point rebuttal to all the academic editor’s and reviewers’ comments is provided below, with the comments displayed in italics and our responses (**A1**-**A32**) in standard font. We also added new data that were not required by the reviewers, as outlined under **A33**, and we modified Figure 6 (**A34**).

We hope that you will find our revisions adequate and the revised manuscript suitable for publication in PLOS ONE.

Sincerely,

Håkan Hedman, corresponding author

*ACADEMIC EDITOR*

*Both reviewers raised concerns about: 1) the lack of data on expression of the co-receptors following Dox treatment and how this relates to physiological levels, and 2) inappropriate statistical analyses. These two points muse be addressed with additional data and statistical analyses. Changes to the interpretation of results may be required as a result. Please address the more minor concerns as well.*

**A1:** Regrettably, it was not possible to directly compare LRIG protein levels between wild-type MEFs and LRIG-inducible Lrig-null MEFs. This was because the wild-type MEFs expressed mouse Lrig proteins, whereas the doxycycline-inducible LRIG alleles encoded the human variants. However, in the revised manuscript, we performed a comparison between the LRIG1 levels obtained in the doxycycline-inducible system with those observed in a series of human cell lines (Supplementary Figure 7). This analysis showed that the LRIG1 levels in doxycycline-inducible MEFs were comparable to those in the human ovarian carcinoma cell line OVSAHO. From this result, we concluded that the LRIG1 levels in the doxycycline-inducible MEFs were physiologically relevant, not supraphysiological. These new results are described in the relevant Results section (Lines 208-218) and Discussion (Lines 282-286).

**A2:** We apologize for the poor statistical analyses and thank the Editor and the reviewers for pointing this out. In the revised manuscript, as suggested by the reviewers, we performed 2-way ANOVAs for all the ligand titration results (Figures 1, 2, 4, and 5). Indeed, these analyses revealed additional significant differences that were not evident from the previously used *t* tests. Most notably, two-way ANOVA showed that LRIG2 also promoted BMP4 signaling (Figures 1 and 5) and that BMP9 and BMP10 signaling was enhanced in *Lrig*-null cells (Figures 4 and 6). In the revised manuscript, these new results are included in the Abstract (Lines 26-28), the Results section (Lines 227, 251-252, and 259-260), and the Discussion (Lines 275, 288, and 294-295).

*Reviewer #1: In their manuscript, Abdullah et al. the regulation of TGFβ signaling by leucine-rich repeats and immunoglobulin-like domains (LRIG) using Lrig-null mouse embryonic fibroblasts (MEFs) with a doxycycline-inducible LRIG1, LRIG2, or LRIG3 overexpression system. Cells were treated with different TGFβ ligands, after treatment, SMAD1/5/8 and SMAD3 phosphorylation, as well as Id1 expression was assessed.*

**A3:** We thank Reviewer #1 for their time in carefully reviewing our manuscript.  *Major points:
1) The authors do not show LRIG1, 2, and 3 protein expression levels before and after doxycycline-induction in their cell system and its correlation with normal/basal levels of LRIG proteins in wild-type MEFs cells. The data are not robust. SMAD-3 phosphorylation is weak in most of the treatments in wild-type cells, however, there is stimulation in the mutant cells treated with or without doxycycline. The authors should explain this discrepancy to validate the use of these cells to study SMAD phosphorylation dependant on LRIGs proteins.*

**A4:** We thank Reviewer #1 for their important comment regarding the induced LRIG protein expression levels. Our response to this question was outlined under **A1**, above.

**A5:** Indeed, treatment of wild-type MEFs with the ligands at their reported ED50 induced rather modest pSmad3 responses (Supplementary Figure 1). In fact, only TGFbeta1 and GDF11 yielded a significant increase in pSmad3 levels (3.5-fold and 1.5-fold, respectively). For our discussion about the ED50s and the decision to use this approach, see **A20** and **A21** below. In the *Lrig*-null cells, we think we observed a responsiveness to TGFbeta1 that was rather similar to the responsiveness shown by wild-type cells (Figure 2, upper row vs. Supplementary Figure 1). However, the response to GDF11 was clearly more robust in the *Lrig*-null cells, with or without doxycycline, (Figure 2, lower row) than in the wild-type cells (Supplementary Figure 1). The reason for this apparent discrepancy in GDF11 responsiveness between *Lrig*-null MEFs and wild-type cells is not known. Notably, however, some responsiveness differences should be expected because we used different MEF lines. The observed clonal variability is the reason why we always used multiple cell lines (≥4) when comparing wild-type and *Lrig*-null cells. In contrast, when analyzing the role of individual LRIG proteins, we used the doxycycline-inducible system, i.e., a single cell line +/- doxycycline. However, in the ligand screen shown in Supplementary Figure 1, we used only one wild-type cell line because the purpose of the experiment was to identify a series of ligands suitable for LRIG studies, not to compare different cell lines or genotypes.

*2) The statistical analysis are not correct in any of the figures. The authors performed t-tests in an experimental design with 2 variables. Two-way ANOVA should be performed.*

**A6:** We thank Reviewer #1 for pointing out the statistical flaws. In the revised manuscript, we performed 2-way ANOVA throughout. Please see also our response under **A2** above.

*3) The authors used different concentrations of doxycycline to analyze LRIG1 and LRIG3 roles after GDF7 stimulation, however, the authors do not show these raw data neither how the different doses of doxycycline affect protein levels of LRIG1 or LRIG3 (Fig. 3).*

**A7:** In the revised manuscript, we added a graph (Supplementary Figure 8) showing the relationships between the doxycycline concentrations and LRIG1 and LRIG3 protein expression levels in the experiment shown in Figure 3. Notably, the inducible LRIG1 protein and the inducible LRIG3 protein both contain an identical FLAG epitope; therefore, their relative expression levels could be directly compared using an anti-FLAG antibody and immunofluorescence. Please also note our discussion about the physiological relevance of the inducible LRIG protein expression level under **A1** above.

*4) It is not clear how robust and reproducible these data are. Experiments should be graphed using individual points, where each point correspond to one independent experiment in all figures.*

**A8:** We have added the individual graphs in Supplementary Figures 3-6.

*5)It is not a clear if Id1 stimulation depends on LRIG proteins, there is dose-response effects in absence of doxycycline (Fig. 5).*

**A9:** Here, we are not sure exactly what Reviewer #1 means. Indeed, we observed BMP and GDF dose-dependent induction of *Id1* both in the absence and in the presence of doxycycline (+/- dox). However, our point here is that doxycycline, i.e., the induction of LRIG expression, enhances the *Id1* response in certain LRIG-BMP/GDF combinations. Maybe some of the confusion derives from our previously ambiguous use of the word “dependency”. In the revised manuscript, we tried to reformulate the relevant paragraphs to clearly indicate that signaling by certain BMPs is regulated by, rather than being strictly dependent on, LRIG proteins. See also our response to the use of the word “dependency”, under **A16**, below.

*Minor comments:
1) Colors and font sizes should be consistent across the figures.*

**A10:** In the revised submission, we tried to be consistent with the colors and font sizes in all the figures.

*2) Protein and gene nomenclature must be modified: proteins should be written in capital letters and genes in lower-case letters and italicized.*

**A11:** We have used the gene and protein names and symbols recommended by the National Center for Biotechnology Information (NCBI). Gene names are written in italics, and protein names are written in standard fonts. Human gene and protein symbols were written in capitals, whereas mouse gene and protein symbols were written with a capital followed by lower case fonts. We regret that this practice might sometimes appear confusing; however, we prefer to stick to these widely adopted international nomenclature conventions.

*3) Graphs and statistical analysis were done with two different version of GraphPad. Please, clarify.*

**A12:** We apologize for the confusion. In the revised manuscript, only GraphPad Prism version 9.0 was used (Lines 165, 169, and 480).

*4) Immunofluorescence images are not available, only the quantification is plotted. A supplementary figure needs be added.*

**A13:** We added a representative figure (Supplementary Figure 9), showing the immunofluorescence images corresponding to the pSmad1/5 quantification of a BMP4 titration experiment.

*Reviewer #2: Abdullah et al. examined signaling of various TGFbeta ligands through the LRIG1-3 co-receptors in murine MEFs. This represents an extension of earlier work by this group. Overall, the manuscript is straightforward and easy to read. Most of my comments are minor in nature. The only ‘major’ concern (#1 below) is the level of expression of the co-receptors when over-expressed, which was not reported (see below). It is possible that additional experiments will be required to address this concern.*

**A14:** We thank Reviewer #2 for their time in carefully reviewing our manuscript.  *Specific comments:

1. The authors do not show expression of the different receptors following Dox treatment. It is important to show the extent to which each receptor was expressed relative to what is seen in wild-type MEFs. The authors imply that the expression level was or might have been supraphysiological (line 238), but they do not show data that speak to this issue. They should. The data in Fig. 3 show (at least for GDF7) that the effect of LRIG1 and LRIG3 on Smad1/5 activation is dependent on the extent of co-receptor expression. We need to see if any of the potentiation (by LRIG3) or attenuation (by LRIG1) occurs when the proteins are expressed at or near physiological levels. The authors claim to address concerns about supraphysiological expression starting on line 238, but this reviewer was not persuaded by their argument. We need to see protein expression and how it relates to what is seen in wild-type MEFs.*

**A15:** We thank Reviewer #2 for this important comment. Our response to this question is outlined under **A1**, above.

*2. Line 19: ‘dependent’ here and elsewhere (‘dependency’, line 184, 233) is not the right word. If something is dependent on something else, it requires it. That is not the case here. LRIG impacts, regulates, influences, etc. signaling by some TGFbeta ligands, but in none of the cases reported here did their signaling depend on any of the LRIG proteins. Please replace the word ‘dependent’ or ‘dependency’ with more accurate terminology.*

**A16:** Indeed, we agree that none of the studied factors is strictly dependent on LRIG proteins for inducing signaling. Hence, we replaced all ‘dependent’, ‘dependency’, ‘dependencies’, and ‘independently’ with other words, such as ‘regulated’ (Lines 18, 57), ‘role of’ (Line 60), ‘regulation’ (Lines 204, 230, 503, and 507), ‘unaffected (Line 278), ‘indifferent’ (Line 306), and ‘not regulated’ (Line 511-512). Figure 6 was also modified accordingly. We hope that these changes and clarifications improved the stringency and clarity of the manuscript.

*3. Line 38: While there may be 33 genes that encode TGFbeta ligand subunits, there are many heterodimers. Therefore, the number of ligands is likely to be far greater than 33.*

**A17:** We thank Reviewer #2 for pointing out this mistake. We have corrected the text accordingly (Lines 40-42).

*4. Line 46: Based on work from the Hill lab, it is more accurate to say that Smad complexes accumulate rather than translocate into the nucleus.*

**A18:** We thank Reviewer #2 for pointing out this important distinction. We revised the manuscript accordingly (Line 49) and added a corresponding reference by Caroline Hill (Line 50).

*5. Line 119: Here and in the associated figures, why normalize with actin rather than with total (unphosphorylated) Smads?*

**A19:** In our experience, actin worked well as the immunoblotting loading control. By using actin as the loading control, we believe that we study the pSmad levels in relation to total cellular protein (or actin), which arguably is likely to represent the “total” signal volume per cell. If we were to normalize against total Smad, instead, we believe that we would rather monitor the pSmad-Smad equilibrium, independently of the total amount of active pSmad. Hence, we think the two normalization procedures represent slightly different points of view, although, in our opinion, they are equally relevant. Therefore, we do not see any strong reason to change the normalization protein from actin to total Smad. Hence, if possible, we would prefer to stick to actin as our loading control.

*6. Line 155: Provide references for the reported EC50s. Are these EC50s specific for these ligands in MEFs? It is surprising that 100 ng/ml was used as the EC50 for GDF11, for example. This seems very high. Also, given the receptor expression in these cells, it is surprising that activin A did not stimulate pSmad3. Perhaps 2 ng/ml is too low in these cells. Ideally, the authors should have done dose-responses for all the ligands used here in these cells. In lieu of asking for these data, I think the authors need to do a better job of justifying the concentrations they used (with references). They might also add some text (to the Discussion) acknowledging that they might have been able to assess LRIG regulation of the other ligands had the latter been tested at different (higher?) concentrations.*

**A20:** We thank Reviewer #2 for this important comment. In the revised manuscript, references for all the ED50 values (EC50 was changed to ED50 in the revision) are provided in Supplementary Table 1.

**A21:** Indeed, it could be argued that we ideally should have tested a wide range of ligand concentrations also in the initial ligand screen. However, we decided to use only one ligand concentration in the initial screen for three main reasons. First, it was a compromise between breadth and depth in the study. Second, the purpose of the study was to investigate the LRIG-dependencies of different TGFbeta family ligands, not to investigate the TGFbeta responsiveness of MEFs, *per se*. Hence, we thought it would be acceptable to miss some ligands, which was also reflected in our choice to not test all possible TGFbeta ligands. Third, because of the promiscuity and “stickiness” among the TGFbeta ligands, we wanted to keep the ligand concentrations relatively low and close to their ED50 to favor specific ligand‒receptor interactions rather than possibly nonspecific and nonphysiological interactions. A brief discussion along these lines has been added to the corresponding Results section (Lines 184-187).

*7. Line 156: There is no description of western blotting for pSmad1/5 in the Methods. At present, western blotting is limited to pSmad3. Please update the Methods.*

**A22:** We thank Reviewer #2 for highlighting this mistake. In the revised manuscript, the description of Western blotting, now consistently named “Immunoblotting”, (Lines 124-144) now also comprises pSmad1/5 (Line 138).

*8. Line 159: The GDF11 data are not convincing here (they are more convincing is subsequent experiments). In Suppl. Fig. 1, only TGFbeta1 convincingly stimulates pSmad3. I don’t see a difference between GDF11 and GDF15 on any of the blots. Also, in the figure, activin should be labeled activin A.*

**A23:** We agree that the screening results in Supplementary Figure 1 may not appear convincing. See also **A5** and **A21** for a discussion about this topic.

**A24:** In response to Reviewer #2’s latter comment, we relabeled ‘activin’ with ‘Activin A’ in Supplementary Figure 1.

*9. Line 170: For the residual Lrig expression, the authors can (and should) look for sequence reads in the floxed exons. If the recombination was complete, there should be no reads for these exons. This information can and should be reported. This seems particularly important for Lrig1.*

**A25:** In the revised manuscript, we added an analysis of the expression of the floxed *Lrig* regions via exon-specific qRT-PCRs (Supplementary Table 5). This analysis revealed some residual expression of the floxed exons. However, the expression levels of the floxed exons were low in the ‘*Lrig*-null’ MEF populations. That is, the *Lrig*-null cells expressed less than 10% of the levels seen in wild-type MEFs. Because these *Lrig*-null MEF lines have not been cloned [Herdenberg et al., 2021], residual expression of floxed exons might indicate the presence of contaminating wild-type cells. However, because of the apparently relatively low fraction of contaminating wild-type cells, we believe that our *Lrig*-null MEF lines are well suited to study LRIG protein function. Information about this qRT‒PCR experiment has been added to the Materials and Methods section (Lines 155-158, Supplementary Table 3) and the Results section (Lines 196-202, Supplementary Table 5).

*10. Line 179: What was the rationale for using IF for pSmad1/5 vs. IB for pSmad3? Why different methods for the two?*

**A26:** We developed a high-throughput analytical platform for the analysis of pSmad1/5 through automated analysis of cellular immunofluorescence [Herdenberg et al., 2021]. However, we were unable to translate this methodology to pSmad3 because of an apparent lack of suitable pSmad3 antibodies. Therefore, we had to use immunoblotting to monitor pSmad3 instead. This information has been added to the revised manuscript (Lines 222-225).

*11. Lines 201-217 (and Figure 5): There is no discussion/mention of the experiment with LRIG2 for BMP2. Also, why wasn’t LRIG2 tested with the other ligands in this experiment?*

**A27:** We regret the omission of LRIG2 with the other ligands in the previous manuscript version. In the revised manuscript, we included LRIG2 in all the analyses (Figures 1, 2, and 5). We also included a discussion about LRIG2, BMP2, and BMP4 (Lines 293-295).

*12. Line 271: I am not convinced that there is a qualitative difference between LRIG2’s effects on BMP2 vs. BMP4. In figure 1, the difference really looks quantitative. I am not certain the correct statistics were used in these experiments. The Methods (line 142) reference t-tests. Given the nature of these experiments (and in Figs. 2, 4, and 5), the authors should be using two-way ANOVAs (or a non-parametric test if the variances are not equal or if they do not log transform their data). In Fig. 1, there is a clear main effect of Dox for BMP4 and BMP6 (maybe even for TGFbeta1). That is, it is clear that LRIG2 is also potentiating their responses as it does for BMP2. The authors should employ the correct statistics and adjust their discussion, as necessary. The correct stats may impact the interpretation of the data in Fig. 4 (BMP9) as well.*

**A28:** We thank Reviewer #2 for pointing out the statistical flaws. Indeed, two-way ANOVA revealed a significant effect of LRIG2 on BMP4 (although not on BMP6 or TGFbeta1). In the revised manuscript, we performed 2-way ANOVAs throughout. For our complete response on this issue, see **A2** above.

Regarding Figure 4, we changed this figure, as described under **A33**, below.

*13. Line 272-273: The differential effect of LRIG1 and LRIG3 on GDF7 signaling is arguably the most novel and interesting of the study. It is disappointing that no mechanistic insight is provided. According to Fig. 6, GDF7 signals preferentially via ALK6. But ALK6 is expressed at low levels in these cells (indeed, as in most cells). Might LRIG1 and LRIG3 differentially affect GDF7 signaling via ALK3 or even ALK2?*

**A29:** We can only agree that it is disappointing that we could not provide any mechanistic insights. However, we think that the described findings can serve as the basis of future studies to illuminate the mechanisms involved. Reviewer #2’s receptor speculations are highly interesting; however, in the revised manuscript, we toned down the speculations about the specific type 1 receptors involved (see **A30**, below).

*14. Lines 285-286: Is the discussion here based on the relative abundance of these receptors (at least at the mRNA level) in these cells? If so, that should be stated explicitly, as there are no functional data here on the roles of these different type I receptors.*

**A30:** Indeed, we feel that the questioned statement might have been too speculative for inclusion in the last paragraph of the Discussion. Instead, we limited the scope of this last Discussion paragraph (Lines 326-330).

*15. Figure 3: Y axis. SMAD is all upper case here but is written as Smad elsewhere. Be consistent.*

**A31:** We thank Reviewer #2 for pointing out this inconsistency. Accordingly, we changed SMAD to Smad in Figure 3.

*16. Figure 6: Activin A should be repositioned. It only used ALK4. It does not signal via ALK7.*

**A32:** We are grateful for this information. In Figure 6, we repositioned Activin A accordingly.

**ADDITIONAL REVISION**

**A33:** In the meantime, when the manuscript was under review, we performed additional experiments to extend the scope of the study, including BMP10 and BMP15. Hence, the MEFs responded to the ED50 of BMP10 but not BMP15. The response to BMP10 was not regulated by individual LRIG proteins, however, it was enhanced in *Lrig*-null cells. These results have been included in the revised manuscript (Abstract, Lines 27-28; Results, Lines 181-183, 233, and 251; Discussion, Lines 288, 303-304, 307, and 312-313; Figures 1, 4, and 6; Supplementary Figure 1).

**A34:** Figure 6 was modified to accommodate the new results and interpretations. Additionally, for clarity, the RNAseq numbers were removed from the figure and the receptors’ Alk names were included instead.
