# Supplementary material for: Ligand-specific regulation of transforming growth factor beta superfamily factors by leucine-rich repeats and immunoglobulin-like domains proteins
Source: PLoS One. 2023 Aug 21;18(8):e0289726. doi: 10.1371/journal.pone.0289726 (PMC10441800; doi:10.1371/journal.pone.0289726)
Supplement: Attachment — Submitted filename: Response to Reviewers 230707.docx [file pone.0289726.s018.docx]

Response to Reviewers

Below follows a point-by-point rebuttal to all the Academic Editor’s and reviewers’ comments, with the comments displayed in italics and our responses (**A1**-**A8**) in standard font.

*ACADEMIC EDITOR:

The authors were responsive to previous feedback. There are just a few minor issues remaining to be addressed. Please see the reviewer comments.*

**A1:** We are pleased to note that only a few minor issues remain to be addressed.

*Reviewer #1: In their revised manuscript, Abdullah et al. addressed most of the previous comments from this reviewer. However, the authors are missing some relevant information in the ms.*

**A2:** We are glad that Reviewer #1 was mostly happy with our revised manuscript.

*1) Post-hoc comparisons used in their statistical analysis (two-way ANOVA) needs to be added.*

**A3:** We thank Reviewer #1 for their statistical advice. Accordingly, we performed Tukey’s multiple comparison test post-hoc to all our two-way ANOVAs. These post-hoc tests did not change any of the significance levels indicated in the previous manuscript. Information about the post-hoc tests were added in the revised manuscript (Lines 165, 230, 458, 469, 495, and 506).

*2) Not all the supplementary figures (S1-S9) are discussed and referred in the text. For example, references to each supplementary figure must be added in lines 211, 218, and 286.*

**A4:** We apologize for this mistake. In the revised manuscript, we referred to all supplementary figures: S1, Line 122; S2, Lines 180 and 250; S3, Lines 212 and 219; S4, Line 459; S5, Line 470; S6, Line 471; S7, Line 485; S8, Line 496; S9, Line 507. We also changed their numbering and order to reflect their order of appearance in the text.

*Reviewer #2: The authors were highly responsive to my prior feedback. It is unfortunate that the results still lack mechanistic insight, but the basic phenomena are interesting and worth reporting.*

**A5:** We are pleased to note Reviewer #2’s positive view on the manuscript.  *I have a few remaining minor comments:

1. There are 9 supplementary figures. These are not presented in order (i.e., reference is made to supplementary figure 9 before 1; 3 is referenced before 2) or are not referenced at all (supplementary figures 4, 5, 6, 7, and 8). Ideally, all supplementary figures should be referenced in the text and/or in the legends, and should be referenced in order of presentation.*

**A6:** We apologize for this mistake. In the revised manuscript we referred to all supplementary figures and changed their numbering and order to reflect their order of appearance in the text.

*2. Line 82: ‘college’ should be ‘colleague’*

**A7:** We thank Reviewer #2 for their careful reading of the manuscript. Accordingly, ‘college’ was changed to ‘colleague’ (Line 82).

*3. Lines 214-218: no figure is referenced in association with the described data. I think this may relate to supplementary figure 7 (though see my first comment).*

**A8:** We apologize for this mistake. In the revised manuscript, we added a reference to Supplementary Figure 3 (Lines 212 and 219).
